# Supplementary material for: Oncogenic Role of Aberrant EZH2 in Hepatoblastoma
Source: bioRxiv. 2025 Aug 1:2025.07.30.667506. Preprint. [Version 1] doi: 10.1101/2025.07.30.667506 (PMC12324517; doi:10.1101/2025.07.30.667506)
Supplement: Supplement 1 [file media-1.pdf]

Supplemental Table 1 - Patient Sample Demographics

Patient ID	Description	Gender	Age at sample collection (years)	Pretext stage	Histology	Predominant histologic subtype	Neoaduvant Chemotherapy prior to acquisition
17	HB	F	5.21	IV	fetal	fetal	Y
18	HB	M	2.21	III	mixed epithelial, fetal, embryonal, minor mesenchymal	mixed	Y
21	HB	M	2.96	IV	Predominant pattern pleomorphic; embryonal; HCC-like	embryonal	Y
23	HB	F	1.51	III	variable histology, no classic fetal/embryonal		Y
24	HB	F	3.81	IV	HCC-like		Y
25	HB	M	2.02	IV	cartilage		Y
27	HB	F	1.73	IV	Crowded fetal, rare multinucleated and pleomorphic, blastemal, pseudoacinar	mixed	Y
28	HB	F	1.58	III	bone, cartilage		Y
29	HB	M	1.88	IV	Metastatic HB, embryonal, undifferentiated morphology	embryonal	Y
30	HB	M	3.40	IV	Crowded fetal, embryonal, blastemal, rare pleomorphic	transition	Y
31	HB	M	2.19	II	mixed epithelial (fetal and embryonal) and mesenchymal without teratoid features	mixed	Y
38	HB	M	2.90	IV	Crowded fetal, blastemal, cholangioblastic	fetal	Y
40	HCC	M	27.08	I	Well-differentiated HCC	HCC	N
41	HB	M	2.06	IV	Crowded fetal, embryonal, blastemal	mixed	Y
42	HB	M	3.09	IV	HCC-like (pleomorphic, steatosis)		Y
43	HB	M	1.51	I	epithelial type, fetal, blastemal	fetal	N
45	HB	M	7.98	III	Epithelial type, macrotrabecular, embryonal, fetal	mixed	Y
46	HB	F	2.64	II	mixed fetal and embryonal, mesenchymal type without teratoid features	mixed	Y
47	HB	M	3.59	III	epithelial type, fetal (crowded), embryonal, blastemal, pleomorphic	mixed	Y
48	HCN-NOS	M	11.90	IV	fetal-like, macro, mild pleomorphism	HCN-NOS	Y
49	HB	M	0.38	III	epithelial type, fetal, embryonal (minor amount), blastemal, pleomorphic	fetal	Y
50	HB	F	0.64	II	Crowded fetal, blastemal, osteoid	fetal	Y
52	HCN-NOS	M	12.15	IV	HCN-NOS	HCN-NOS	Y
53	HB	F	2.20	IV	Crowded fetal, embryonal, blastemal	embryonal	Y
60	HB	M	3.57	II	Epithelial type, predominantly embryonal with fetal and blastemal components	embryonal	Y
62	HB				Metastatic HBL, mesenchymal, osteoid	mesenchymal	Y
64	HB	M	1.63	IV	Metastatic HBL, mesenchymal, osteoid	mesenchymal	Y
66	HB	F	3.94	III	epithelial type, mixed fetal and embryonal with focal macrotrabecular and minor blastemal	mixed	Y
67	HB	F	5.04	II	epithelial type, mixed embryonal and fetal blastemal, pleomorphic	mixed	Y
69	HB	F	0.84	III	Crowded fetal	fetal	Y
70	HB	M	4.00	II	Metastatic HB, epithelial type, predominantly embryonal with crowded-fetal and minimal blastemal component	embryonal	Y
71	HCN-NOS	M	7.56	IV	HCN-NOS	HCN-NOS	Y
73	HB	M	1.19	IV	epithelial, predominantly fetal, only small embryonal	fetal	Y

74	HB	F	3.35	IV	Crowded fetal, embryonal, blastemal, mild pleomorphic	embryonal	Y
75	HCN-NOS	M	16.00	III	HCN-NOS	HCN-NOS	Y
76	HB	F	1.08	III	fetal	fetal	Y
77	HCN-NOS	M	13.64	IV	HCN-NOS	HCN-NOS	Y
79	HCC	F	16.44	IV	HCC	HCC	Y
80	HB	F	0.63	II	Epithelial, fetal	fetal	Y
81	HB	M	4.65	II	Crowded fetal, embryonal	embryonal	Y
82	HB	F	3.70	IV	Pleomorphic, HCC-like areas		Y
83	HB	F	1.88	III	epithelial type, embryonal, pleomorphic (poorly differentiated), SCUD, mesenchymal type without teratoid features	mixed	Y
84	HCC	M	13.20	I	HCC, moderately differentiated	HCC	N
85	HB	M	6.59	I	epithelial type, embryonal, fetal (crowded), cholangioblastic and blastemal, focal mesenchymal	mixed	Y
86	HB	M	3.29		Metastatic HB; blastemal with focal mesenchymal present		Y
87	HB	F	1.31	III	mixed epithelial and mesenchymal, fetal pattern (mitotically inactive), mesenchymal without teratoid	fetal	Y
91	HB	F	3.02	II	mixed epithelial and mesenchymal, fetal (mitotically active), embryonal, pleomorphic (poorly differentiated)	mixed	Y
92	HB	F	0.82	II	epithelial type, fetal (mitotically active), blastemal, with cholangioblastic differentiation		Y
94	HB	F	0.99	IV	epithelial type, fetal pattern, mesenchymal	fetal	Y
96	UES	F	6.64	II	undifferentiated embryonal sarcoma	UES	Y
97	HB	F	2.83	IV	fetal	Fetal	Y
99	UES	F	6.91	II	undifferentiated embryonal sarcoma	UES	Y
105	HCC	M	10.96		moderately differentiated	HCC	N
108	HCN-NOS	M	11.50	III	Pleomorphic fetal of HB overlap with HCN-NOS	fetal	Y
129	HB	M	4.43	II	mixed fetal, crowded fetal and blastemal with focal pleomorphic and cholangioblastic differentiation	mixed	Y
130	HB	M	5.20	I	crowded fetal, embryonal, mixested with blastemal	mixed	Y

Supplemental Table 2- Antibodies and real time PCR primers

Antibodies	Dilution	Company	Product Number	Publication
IHC/IF				
EZH2 (IHC, IP-Western)	1:200	Cell Signaling	3147	PMID: 35301492
SUZ12	1:1,000	Cell Signaling	3737	PMID: 36612203
EED	1:200	Cell Signaling	85322	PMID: 36428492
Alexa Fluor 488 goat anti-mouse IgG	1:2,000	Invitrogen	A11001	
Alexa Fluor 555 goat anti-rabbit IgG	1:2,000	Invitrogen	A21428	

Gene Primers	Company	Product Number
EZH2	Qiagen	PPH02880A
SUZ12	Qiagen	PPH17208A
EED	Qiagen	PPH23422
CTNNB1	Qiagen	PPH00643F
Ki67	Qiagen	PPH01024E
GAPDH	Qiagen	PPH00150F
AURKB	Qiagen	PPH21059F
GPC3	Qiagen	PPH11457B
STAT3	Qiagen	PPH00708F
CDH1	Qiagen	PPH00135F
TGFβ	Qiagen	PPH00508A
MYC	Qiagen	PPH00100B
